# Supplementary material for: Rolling Circle cDNA Synthesis Uncovers Circular RNA Splice Variants
Source: Int J Mol Sci. 2019 Aug 16;20(16):3988. doi: 10.3390/ijms20163988 (PMC6721031; doi:10.3390/ijms20163988)
Supplement: Supplementary file 1 [file ijms-20-03988-s001.zip › Das et al. Supplimentary files/Das et al. Supplementary text & Figure_Revised.pdf]

Rolling Circle cDNA Synthesis Uncovers Circular RNA Splice Variants

Aniruddha Das[1,2], Pranita K. Rout[1], Myriam Gorospe[3], and Amaresh C. Panda[1,*]

[1] Institute of Life Sciences, Nalco Square, Bhubaneswar, Odisha, India;

[2] School of Biotechnology, KIIT University, Bhubaneswar, India;

[3] Laboratory of Genetics and Genomics, National Institute on Aging, NIH, Baltimore, Maryland, USA.

[*]Correspondence: amaresh.panda@ils.res.in; Tel: +91 674 230 4314; Fax: +91 674 230 0728

Supplementary Materials: Figure S1. Validation of circRNA expression using divergent primers. **Figure S2.** Splice variants of hsa_circ_0001566. **Figure S3.** Splice variants of hsa_circ_0007822. **Figure S4.** Splice variants of hsa_circ_0084615. **Figure S5.** Splice variants of hsa_circ_0009156. **Figure S6.** Splice variants of hsa_circ_0071410. **Figure S7.** Potential miRNA targets of circRNA splice variants. **Figure S8.** Potential RBPs associated with circRNA splice variants. **Supplementary Table S1.** Sequences of the DNA primers used to in this study. **Supplementary Table S2.** Gene Ontology analysis for target genes of miRNAs associated with has_circ_007127 sequence reported in circBase and the splice variants.

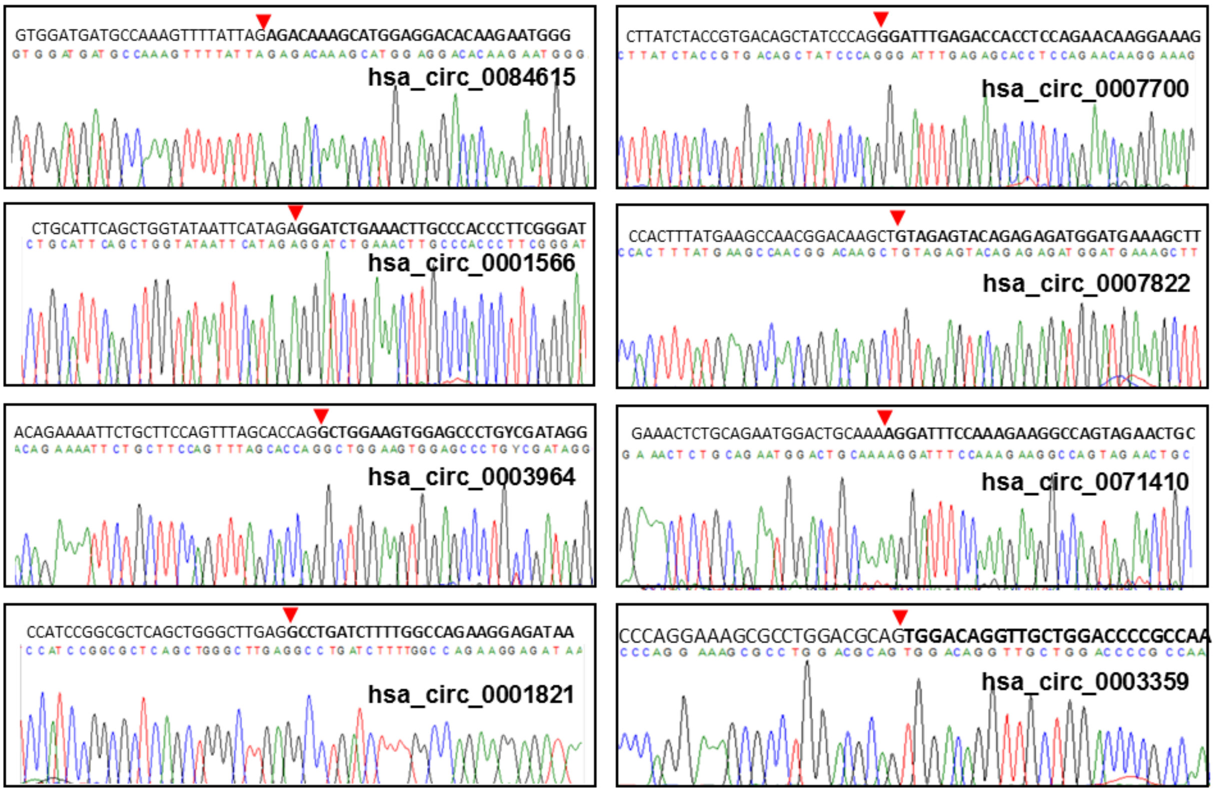

Figure S1. Validation of circRNA expression using divergent primers. RT-PCR products amplified with divergent primers were purified and verified by Sanger sequencing to confirm the identity of the backsplice junction sequences.

Das, et al, Fig. S2

A

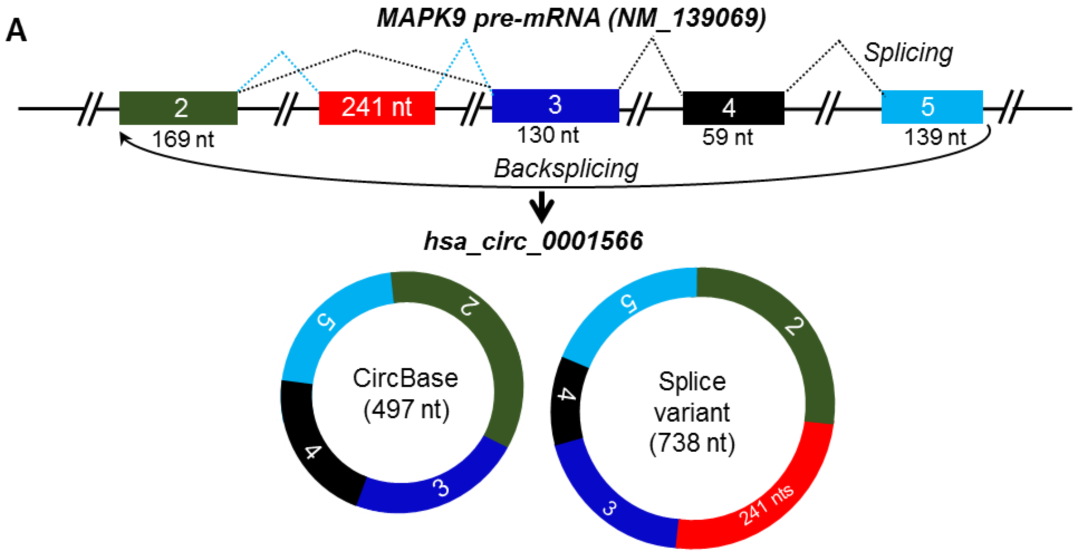

B

hsa_circ_0001566 **(CircBase: 497 nt)**

GGATCTGAAACTTGCCCACCCTTCGGGATATTGCAGGACGCTGCATCATGAGCGACAGTAAAT
GTGACAGTCAGTTTTATAGTGTGCAAGTGGCAGACTCAACCTTCACTGTCCTAAAACGTTACCA
GCAGCTGAAACCAATTGGCTCTGGGGCCCAAGGGATTGTTTGTGCTGCATTTGATACAGTTCTT
GGGATAAATGTTGCAGTCAAGAAACTAAGCCGTCCTTTTCAGAACCAAACTCATGCAAAGAGAG
CTTATCGTGAACTTGTCCTCTTAAAATGTGTCAATCATAAAAATATAATTAGTTTGTTAAATGTGT
TTACACCACAAAAAACTCTAGAAGAATTTCAAGATGTGTATTTGGTTATGGAATTAATGGATGCT
AACTTATGTCAGGTTATTCACATGGAGCTGGATCATGAAAGAATGTCCTACCTTCTTTACCAGAT
GCTTTGTGGTATTAAACATCTGCATTCAGCTGGTATAATTCATAGA

hsa_circ_0001566 **(Splice variant: 738 nt)**

GGATCTGAAACTTGCCCACCCTTCGGGATATTGCAGGACGCTGCATCATGAGCGACAGTAAAT
GTGACAGTCAGTTTTATAGTGTGCAAGTGGCAGACTCAACCTTCACTGTCCTAAAACGTTACCA
GCAGCTGAAACCAATTGGCTCTGGGGCCCAAGGGATTGTTTG**GCGCCATCATTGCAGTGCCTT**
GCCATTCATTGGCAGGATGAGGCCAGTCAGTGTCATTGTCACTCCATGGATTAACCAGGAAG
GGATGCTGTGTGAGGGGTTGGGCTGGCCATCTCTTCCTTGGTGTCTCACTTCAACTGTGTGTC
CAGCCCAGAACGTCAGAATCGTTAGTCTGACCCTGAACGTTAGTCTCTTGCATGGTAAGACG
GTCAGCCGCCGCAGCATCACGGGCCAGCTCAAGTGCTGCATTTGATACAGTTCTTGGGATAA
ATGTTGCAGTCAAGAAACTAAGCCGTCCTTTTCAGAACCAAACTCATGCAAAGAGAGCTTATCG
TGAACTTGTCCTCTTAAAATGTGTCAATCATAAAAATATAATTAGTTTGTTAAATGTGTTTACACC
ACAAAAAACTCTAGAAGAATTTCAAGATGTGTATTTGGTTATGGAATTAATGGATGCTAACTTAT
GTCAGGTTATTCACATGGAGCTGGATCATGAAAGAATGTCCTACCTTCTTTACCAGATGCTTTG
TGGTATTAAACATCTGCATTCAGCTGGTATAATTCATAGA

Figure S2. Splice variants of *hsa_circ_0001566*. **(A)** Schematic representation of the *MAPK9* pre-mRNA and the biogenesis of *hsa_circ_0001566* splice variants produced by alternative splicing. Boxes and straight lines represent exons and introns, respectively. The black dotted lines represent splicing while blue dotted lines represent alternative splicing. **(B)** Spliced full-length sequences of circBase and splice variants of *hsa_circ_0001566.*

A

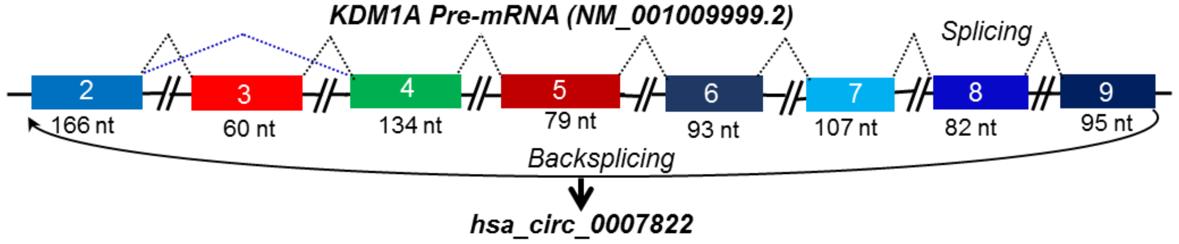

B

***hsa_circ_0007822* (circBase: 816 nt)**

GTAGAGTACAGAGAGATGGATGAAAGCTTGGCCAACCTCTCAGAAGATGAGTATTATTCAGAAGAA
GAGAGAAATGCCAAAGCAGAGAAGGAAAAGAAGCTTCCCCCACCACCCCCTCAAGCCCCACCTGA
GGAAGAAAATGAAAGTGAGCCTGAAGAACCATCGG**GGCAAGCAGGAGGACTTCAAGACGACAGT**
TCTGGAGGGTATGGAGACGGCCAAGCATCAGGTGTGGAGGGCGCAGCTTTCCAGAGCCGACTTC
CTCATGACCGGATGACTTCTCAAGAAGCAGCCTGTTTTCCAGATATTATCAGTGGACCACAACAGA
CCCAGAAGGTTTTTCTTTTCATTAGAAACCGCACACTGCAGTTGTGGTTGGATAATCCAAAGATTCA
GCTGACATTTGAGGCTACTCTCCAACAATTAGAAGCACCTTATAACAGTGATACTGTGCTTGTCCAC
CGAGTTCACAGTTATTTAGAGCGTCATGGTCTTATCAACTTCGGCATCTATAAGAGGATAAAACCCC
TACCAACTAAAAAGACAGGAAAGGTAATTATTATAGGCTCTGGGGTCTCAGGCTTGGCAGCAGCTC
GACAGTTACAAAGTTTTGGAATGGATGTCACACTTTTGGAAGCCAGGGATCGTGTGGGTGGACGA
GTTGCCACATTTCGCAAAGGAAACTATGTAGCTGATCTTGGAGCCATGGTGGTAACAGGTCTTGGA
GGGAATCCTATGGCTGTGGTCAGCAAACAAGTAAATATGGAACTGGCCAAGATCAAGCAAAAATGC
CCACTTTATGAAGCCAACGGACAAGCT

***hsa_circ_0007822* (Splice variant: 756 nt)**

GTAGAGTACAGAGAGATGGATGAAAGCTTGGCCAACCTCTCAGAAGATGAGTATTATTCAGAAGAA
GAGAGAAATGCCAAAGCAGAGAAGGAAAAGAAGCTTCCCCCACCACCCCCTCAAGCCCCACCTGA
GGAAGAAAATGAAAGTGAGCCTGAAGAACCATCGGGTGTGGAGGGCGCAGCTTTCCAGAGCCGA
CTTCCTCATGACCGGATGACTTCTCAAGAAGCAGCCTGTTTTCCAGATATTATCAGTGGACCACAA
CAGACCCAGAAGGTTTTTCTTTTCATTAGAAACCGCACACTGCAGTTGTGGTTGGATAATCCAAAGA
TTCAGCTGACATTTGAGGCTACTCTCCAACAATTAGAAGCACCTTATAACAGTGATACTGTGCTTGT
CCACCGAGTTCACAGTTATTTAGAGCGTCATGGTCTTATCAACTTCGGCATCTATAAGAGGATAAAA
CCCCTACCAACTAAAAAGACAGGAAAGGTAATTATTATAGGCTCTGGGGTCTCAGGCTTGGCAGCA
GCTCGACAGTTACAAAGTTTTGGAATGGATGTCACACTTTTGGAAGCCAGGGATCGTGTGGGTGGA
CGAGTTGCCACATTTCGCAAAGGAAACTATGTAGCTGATCTTGGAGCCATGGTGGTAACAGGTCTT
GGAGGGAATCCTATGGCTGTGGTCAGCAAACAAGTAAATATGGAACTGGCCAAGATCAAGCAAAA
ATGCCCACTTTATGAAGCCAACGGACAAGCT

Figure S3. Splice variants of *hsa_circ_0007822*. (A) Schematic representation of the *hsa_circ_0007822* splice variants biogenesis from *KDM1A* pre-mRNA. Boxes and straight lines represent exons and introns, respectively. The black dotted lines represent splicing while blue dotted lines represent alternative splicing. **(B)** Spliced full-length sequences of splice variants of *hsa_circ_0007822*. The text colors correspond to the colors of the exon boxes in panel A.

A

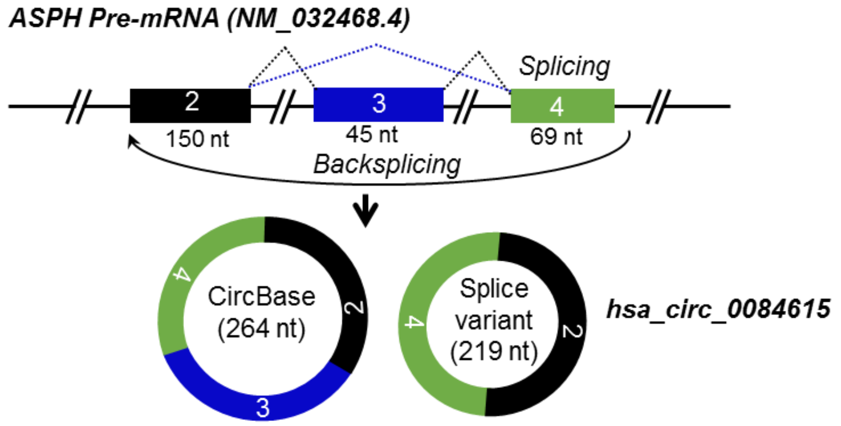

B

***hsa_circ_0084615* (CircBase: 264 nt)**
AGACAAAGCATGGAGGACACAAGAATGGGAGGAAAGGCGGACTCTCAGGAACTTCATTCTTCAC
GTGGTTTATGGTGATTGCATTGCTGGGCGTCTGGACATCTGTAGCTGTCGTTTGGTTTGATCTTG
TTGACTATGAGGAAGTTCTAG**CCAAAGCAAAGGACTTCCGTTATAACTTATCAGAGGTGCTTCA**
AGGAAAACTAGGAATCTATGATGCTGATGGTGATGGAGATTTTGATGTGGATGATGCCAAAGTTT
TATTAG

***hsa_circ_0084615* (Splice variant: 219 nt)**
AGACAAAGCATGGAGGACACAAGAATGGGAGGAAAGGCGGACTCTCAGGAACTTCATTCTTCAC
GTGGTTTATGGTGATTGCATTGCTGGGCGTCTGGACATCTGTAGCTGTCGTTTGGTTTGATCTTG
TTGACTATGAGGAAGTTCTAGGAAAACTAGGAATCTATGATGCTGATGGTGATGGAGATTTTGAT
GTGGATGATGCCAAAGTTTTATTAG

Figure S4. Splice variants of *hsa_circ_0084615*. (A) Schematic representation of the *hsa_circ_0084615* splice variants biogenesis from *ASPH* pre-mRNA. Boxes and straight lines represent exons and introns, respectively. The black dotted lines represent splicing while blue dotted lines represent alternative splicing. **(B)** Spliced full-length sequences of splice variants of *hsa_circ_0084615*. The text colors correspond to the colors of the exon boxes in panel A.

A

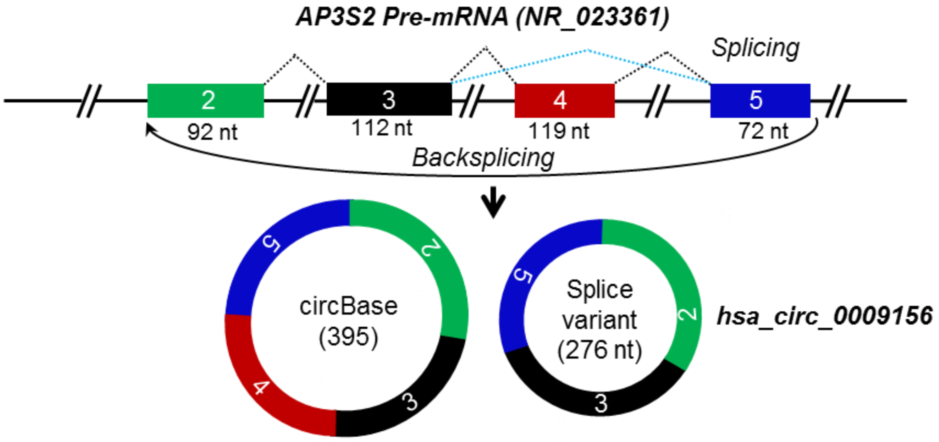

B

hsa_circ_0009156 **(circBase: 395 nt)**
CCAGAAGAAATTCAACAGCAGATTGTTCGAGAGACTTTCCATCTAGTCCTCAAGCGGGATGACAA
CATCTGTAACTTCTTGGAGGGTGGAAGTTTGATTGGTGGCTCTGACTACAAACTGATCTACCGGC
ACTATGCTACCCTCTACTTTGTATTTTGTGTGGATTCCTCAGAGAGTGAACTTGGAATCTTGGACC
TCATCCAG**CTTAAAAAAAGAGAGAGGAAGAAAAGCCAGTGCAACACCCTACAAACTGGAACA**
CTAGCTCTTTGGGAACAAGGACCTGACATCAGAACAAGAAGGCTATAAGTTCCCAAACCTTAA
AAGTTTTTGTGGAAACTCTGGATAAGTGTTTCGAAAATGTGTGTGAATTGGATTTGATCTTCCATA
TGGATAAG

hsa_circ_0009156 **(Splice variant: 276 nt)**
CCAGAAGAAATTCAACAGCAGATTGTTCGAGAGACTTTCCATCTAGTCCTCAAGCGGGATGACAA
CATCTGTAACTTCTTGGAGGGTGGAAGTTTGATTGGTGGCTCTGACTACAAACTGATCTACCGGC
ACTATGCTACCCTCTACTTTGTATTTTGTGTGGATTCCTCAGAGAGTGAACTTGGAATCTTGGACC
TCATCCAGGTTTTTGTGGAAACTCTGGATAAGTGTTTCGAAAATGTGTGTGAATTGGATTTGATCT
TCCATATGGATAAG

Figure S5. Splice variants of *hsa_circ_0009156*. (A) Schematic representation of the biogenesis of the splice variants of *hsa_circ_0009156* from precursor *AP3S2* pre-mRNA. Boxes and straight lines represent exons and introns, respectively. Thee black dotted lines represent splicing while blue dotted lines represent alternative splicing. **(B)** Spliced full-length sequences of splice variants of *hsa_circ_0009156*. The text color corresponds to the color of the exon box in panel A.

A

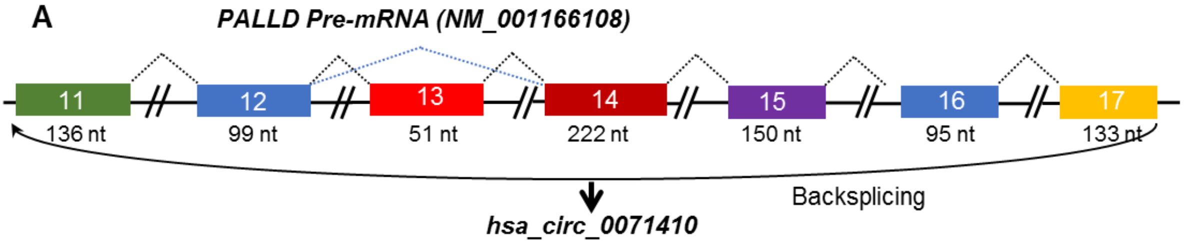

B

***hsa_circ_0071410* (circBase: 886 nt)**

AGGATTTCCAAAGAAGGCCAGTAGAACTGCTAGAATAGCCTCCGATGAGGAAATTCAAGGCACA
AAGGATGCTGTTATTCAAGACCTGGAACGAAAACTTCGCTTCAAGGAGGACCTCCTGAACAATG
GCCAGCCGAGGTTAACATACGAAGAAAGAATGGCTCGTCGACTGCTAGGTGCTGACAGTGCAA
CTGTCTTTAATATTCAGGAGCCAGAAGAGGAAACAGCTAATCAG**GACATTGGTTCTCCTCATGC**
TTCTGTAGGGAGTCCTCTGGATGGTCAAAAGGAATACAAAGTCTCCAGCTGTGAACAGAGACT
CATCAGTGAAATAGAGTACAGGCTAGAAAGGTCTCCTGTGGATGAATCAGGTGATGAAGTTCAG
TATGGAGATGTGCCTGTGGAAAATGGAATGGCACCATTCTTTGAGATGAAGCTGAAACATTACA
AGATCTTTGAGGGAATGCCAGTAACTTTCACATGTAGAGTGGCTGGAAATCCAAAGCCAAAGAT
CTATTGGTTTAAAGATGGGAAGCAGATCTCTCCAAAGAGTGATCACTACACCATTCAAAGAGATC
TCGATGGGACCTGCTCCCTCCATACCACAGCCTCCACCCTAGATGATGATGGGAATTATACAAT
TATGGCTGCAAACCCTCAGGGCCGCATCAGTTGTACTGGACGGCTAATGGTACAGGCTGTCAA
CCAAAGAGGTCGAAGTCCCCGGTCTCCCTCAGGCCATCCTCATGTCAGAAGGCCTCGTTCTAG
ATCAAGGGACAGTGGAGACGAAAATGAACCAATTCAGGAGCGATTCTTCAGACCTCACTTCTTG
CAGGCTCCTGGAGATCTGACTGTTCAAGAAGGAAAACTCTGCAGAATGGACTGCAAA

***hsa_circ_0071410* (Splice variant: 835 nt)**

AGGATTTCCAAAGAAGGCCAGTAGAACTGCTAGAATAGCCTCCGATGAGGAAATTCAAGGCACA
AAGGATGCTGTTATTCAAGACCTGGAACGAAAACTTCGCTTCAAGGAGGACCTCCTGAACAATG
GCCAGCCGAGGTTAACATACGAAGAAAGAATGGCTCGTCGACTGCTAGGTGCTGACAGTGCAA
CTGTCTTTAATATTCAGGAGCCAGAAGAGGAAACAGCTAATCAGGAATACAAAGTCTCCAGCTG
TGAACAGAGACTCATCAGTGAAATAGAGTACAGGCTAGAAAGGTCTCCTGTGGATGAATCAGGT
GATGAAGTTCAGTATGGAGATGTGCCTGTGGAAAATGGAATGGCACCATTCTTTGAGATGAAGC
TGAAACATTACAAGATCTTTGAGGGAATGCCAGTAACTTTCACATGTAGAGTGGCTGGAAATCCA
AAGCCAAAGATCTATTGGTTTAAAGATGGGAAGCAGATCTCTCCAAAGAGTGATCACTACACCAT
TCAAAGAGATCTCGATGGGACCTGCTCCCTCCATACCACAGCCTCCACCCTAGATGATGATGG
GAATTATACAATTATGGCTGCAAACCCTCAGGGCCGCATCAGTTGTACTGGACGGCTAATGGTA
CAGGCTGTCAACCAAAGAGGTCGAAGTCCCCGGTCTCCCTCAGGCCATCCTCATGTCAGAAGG
CCTCGTTCTAGATCAAGGGACAGTGGAGACGAAAATGAACCAATTCAGGAGCGATTCTTCAGAC
CTCACTTCTTGCAGGCTCCTGGAGATCTGACTGTTCAAGAAGGAAAACTCTGCAGAATGGACTG
CAAA

Figure S6. Splice variants of *hsa_circ_0071410*. (A) Schematic representation of the biogenesis of *hsa_circ_0071410* splice variants from *PALLD* pre-mRNA. Boxes and straight lines represent exons and introns, respectively. Thee black dotted lines represent splicing while blue dotted lines represent alternative splicing. **(B)** Spliced full-length sequences of splice variants of *hsa_circ_0071410*. The text colors correspond to the colors of the exon boxes in panel A.

hsa_circ_0009156 (miRNA sites)	
Splice variant (276 nt)	circBase (395 nt)
hsa-miR-6503-3p (1)	hsa-miR-6503-3p (1)
hsa-miR-3692-3p (1)	hsa-miR-3692-3p (1)
hsa-miR-490-3p (1)	hsa-miR-320e (1)
	hsa-miR-5195-3p (1)
	hsa-miR-145-5p (1)
	hsa-miR-186-3p (1)

Figure S7. Potential miRNA targets of circRNA splice variants. MicroRNAs predicted by miRDB web tool to target splice variants of *hsa_circ_0009156*.

Das et al, Fig S8

A

hsa_circ_0009156 (RBP sites)	
**Splice variant (276 nt)**	**circBase (395 nt)**
PTBP1 (13)	PTBP1 (16)
SNRNP70 (2)	SNRNP70 (4)
SRSF1 (7)	SRSF1 (5)
SRSF10 (4)	SRSF10 (9)
SRSF3 (29)	SRSF3 (40)
SRSF5 (6)	SRSF5 (7)
SRSF7 (5)	SRSF7 (7)
TRA2B (9)	TRA2B (19)
YBX2 (3)	YBX2 (7)
	CNOT4 (1)
	ENOX1 (2)
	FMR1 (3)
	FXR2 (1)
	HNRNPA1 (3)
	HNRNPH1 (4)
	HNRNPK (1)
	HNRNPL (3)
	IGF2BP2 (2)
	IGF2BP3 (2)
	KHDRBS1 (5)
	KHDRBS2 (4)
	KHDRBS3 (6)
	PABPC1 (7)
	PABPC4 (7)
	PABPN1 (9)
	SART3 (7)
	SRSF9 (1)
	TRA2A (4)

B

hsa_circ_0084615 (RBP sites)	
**Splice variant (219 nt)**	**circBase (264 nt)**
CUG-BP (16)	CUG-BP (17)
G3BP2 (1)	G3BP2 (2)
MBNL1 (14)	MBNL1 (13)
SRSF1 (13)	SRSF1 (12)
SRSF2 (17)	SRSF2 (16)
SRSF3 (20)	SRSF3 (14)
SRSF5 (7)	SRSF5 (5)
TRA2B (4)	TRA2B (3)
	DAZAP1 (3)
	MSI1 (2)
	PABPC5 (2)
	SRSF7 (1)

Figure S8. Potential RBPs associated with circRNA splice variants. Selected list of RBPs predicted by RBPmap web tool to target splice variants of *hsa_circ_0009156* (A), and *hsa_circ_0084615* (B).
